# Association between glycolipids and risk of obstructive sleep apnea: A population-based study

**DOI:** 10.3389/fnut.2023.974801

**Published:** 2023-03-16

**Authors:** Murui Zheng, Xueru Duan, Huanning Zhou, Weidi Sun, Guoqiang Sun, Jianying Chen, Xiuyi Wu, Sijing Rong, Jun Huang, Wengjing Zhao, Hai Deng, Xudong Liu

**Affiliations:** ^1^Department of Community Health, Guangzhou Center for Disease Control and Prevention, Guangzhou, China; ^2^Department of Epidemiology, School of Public Health, Sun Yat-sen University, Guangzhou, China; ^3^School of Public Health, Guangdong Pharmaceutical University, Guangzhou, China; ^4^Guangzhou Yuexiu District Center for Disease Control and Prevention, Guangzhou, China; ^5^School of Public Health and Women’s Hospital, Zhejiang University School of Medicine, Hangzhou, China; ^6^Baiyun Street Community Health Service Center, Guangzhou, China; ^7^Nancun Town Community Health Service Center, Guangzhou, China; ^8^Guangzhou Haizhu District Center for Disease Control and Prevention, Guangzhou, China; ^9^Department of Geriatrics, Guangdong Provincial People’s Hospital, Institute of Geriatrics, Guangdong Academy of Medical Science, Guangzhou, China; ^10^School of Public Health and Emergency Management, Southern University of Science and Technology, Shenzhen, China; ^11^Department of Cardiology, Guangdong Provincial People’s Hospital, Guangdong Cardiovascular Institute, Guangdong Academy of Medical Science, Guangzhou, China

**Keywords:** lipid, blood glucose, obstructive sleep apnea, risk factor, biomarker

## Abstract

**Background:**

This study aimed to investigate the associations between multiple glycolipid biomarkers and the risk of obstructive sleep apnea (OSA).

**Methods:**

Participants (10,286) aged from 35 to 74 years old were included in this cross-sectional study from the baseline survey of the Guangzhou Heart Study. OSA was ascertained using both Berlin Questionnaire and STOP-BANG Questionnaire. Fasting blood samples were collected from each participant; fasting blood glucose (FBG) and serum concentrations of high-density lipoprotein cholesterol (HDL-CH), low-density lipoprotein cholesterol (LDL-CH), total cholesterol (TC), and triglyceride (TG) were determined. Odds ratio (OR) with 95% confidence interval (CI) was calculated using the multivariate logistic regression model after adjustment for covariates.

**Results:**

Of the participants included, 15.56% were categorized into the pre-OSA group, and 8.22% into the OSA group. When comparing the highest with the lowest quartiles, HDL-HC was associated with a 22% (OR: 0.78, 95% CI: 0.65–0.94) and 41% (OR: 0.59, 95% CI: 0.45–0.78) reduced risk of pre-OSA and OSA, triglyceride was associated with a 32% (OR 1.32, 95% CI 1.08–1.60) and a 56% (OR 1.56, 95% CI 1.18–2.07) increased risk of pre-OSA and OSA, and FBG was associated with a 1.37-fold (95% CI 1.13–1.67) risk of pre-OSA and 1.38-fold (95% CI 1.03–1.85) risk of OSA. A significant exposure-response trend was observed for HDL-HC, TG, and FBG with both OSA and Pre-OSA (all *p* < 0.05). No significant association of LDL-CH and TC with the risk of both pre-OSA and OSA was observed.

**Conclusion:**

The findings suggest that serum HDL-CH was inversely associated with OSA risk, while elevated serum TG and FBG could increase the risk of OSA. Healthy glycolipid metabolism warrants more attention in the field of OSA prevention.

## Introduction

Obstructive sleep apnea (OSA) is a common sleep disorder characterized by recurrent episodes of upper airway obstruction and hypopnea during sleep [1]. It is an adverse health condition that has become increasingly prevalent worldwide [2]. The overall number of affected adults has reached approximately 1 billion [3]. In the United States, the estimated prevalence of OSA ranged from 7 to 12% among men aged 30–49 years old and was even higher as the age went up [4]. China was considered to have the largest number of individuals with OSA among 16 countries [3]. OSA was well recognized as an independent risk factor for multifactorial consequences including cardiovascular diseases, cognitive impairment, [5, 6].

Previous studies have shown that OSA is associated with multiple risk factors, including a low level of physical activity, obesity, and metabolic syndrome [7, 8]. OSA can be both a sleep disorder and a heterogeneous metabolic disorder [9]. Glycolipid biomarkers are considerable screening tools in many chronic diseases [10, 11], the level of which is also considered to be independently associated with the risk of OSA. Pathogenic pathways of the association are consist of activation of the sympathetic nervous system, changes in hypothalamic–pituitary–adrenal axis activity, and formation of reactive oxygen species, etc. [12, 13]. Previous studies have shown that individuals with OSA had a higher prevalence of elevated total cholesterol (TC) and triacylglycerol (TG) [14], and a lower level of high-density lipoprotein cholesterol (HDL-CH) [15]. Dyslipidemia and diabetes, two types of diseases resulting from the dysfunction of glycolipids, were also observed to be associated with OSA in several reports [8, 16, 17]. However, no conclusive results have been found in research on the associations between glycolipid biomarkers and OSA up till now. In a multiethnic study among American people, lower HDL-CH was associated with a higher apnea-hypopnea index (AHI), the key metric used to define OSA severity [18]. In contrast, a remarkable association with AHI was observed in low-dense lipoprotein cholesterol (LDL-CH) but not in HDL-CH among Chinese people [19]. Besides, data from the Heart Institute in America did not present a significant association between HDL-CH and OSA [20].

Therefore, this study aimed to assess the association between glycolipid biomarkers and the risk of OSA using data from the Guangzhou Heart Study (GZHS).

## Method

### Setting and subjects

Participants in this cross-sectional study were recruited from baseline survey of the Guangzhou Heart Study (GZHS), which recruited 12,013 individuals aged 35 years old and above using a randomized multistage cluster sampling between July 2015 and August 2017. Detailed information of the cohort has been reported elsewhere [7, 21–23]. The inclusion criteria for this study were: (1) Guangzhou permanent residents, (2) aged 35 years old and above; (3) having lived in the selected communities for at least 6 months before being involved into the study. Those who were aged 75 years old and above, were pregnant or lactating women, were non-permanent residents in Guangzhou, had mental or cognitive disorders including dementia, disturbance of understanding and deaf-mutters, had mobility difficulties including high paraplegia, and had any cancer history, were excluded.

Finally, a total of 10,826 individuals from the GZHS baseline survey were involved for further analysis. This study was approved by the Ethical Review Committee for Biomedical Research, School of Public Health, Sun Yat-sen University, and Ethics Committee of Guangzhou Centre for Disease Control and Prevention. It was conducted in accordance with the Declaration of Helsinki. Written informed consent was obtained from all participants.

### Ascertainment of OSA

Obstructive sleep apnea was ascertained by Berlin Questionnaire (BQ) and STOP-BANG Questionnaire (SBQ), which are both widely used as screening tools for identifying OSA. The Chinese versions of both questionnaires had been proved to have superior predictive validity and reliability [24–27]. Berlin Questionnaire contains ten questions in three categories: snoring and cessation of breathing (category 1), symptoms of excessive daytime sleepiness (category 2), body mass index (BMI), and hypertension (category 3). High risk in category 1 and category 2 was defined by persistent symptoms (> 3–4 times/week) in at least two questions of each category, and high risk in category 3 was defined by the history of hypertension or BMI higher than 30 kg/m^2^. When two or more categories were considered positive, it indicated a high risk for OSA, otherwise a low-risk for OSA [28]. The SBQ included eight questions with dichotomous (yes/no) answers. The questions referred to snoring, fatigue, observed apnea, high blood pressure (or treatment for it), body mass index (BMI > 35 kg/m^2^), age (> 50 years old), neck circumference (> 40 cm), and gender (male). For each question, answering “yes” scores 1 and a “no” response scores 0. Subjects scoring three or more were considered to be at a high risk of OSA, otherwise were as at a low risk of OSA [24]. Then, participants who were assessed as having a high risk of OSA by both BQ and SBQ were classified into the OSA group, those who were assessed as having a lower risk of OSA by both BQ and SBQ were classified into the non-OSA group, and the remain participants were classified in the pre-OSA group.

### Measurements of glycolipid biomarkers

Fasting blood samples were collected in the morning from each participant at the baseline survey and then detected within 4 h after collection in a qualified third-party medical laboratory (Guangzhou KingMed Diagnostics Group Co., Ltd). Fasting blood glucose (FBG) and serum lipids, including high-density lipoprotein cholesterol (HDL-CH), low-density lipoprotein cholesterol (LDC-CH), total cholesterol (TC), and triglyceride, were detected. 5% samples were randomly selected for parallel double-sample detection, and the results showed that the detection results were reliable.

### Measurements of covariates

A face-to-face interview approach and a medical examination were adopted to collect information. A structured questionnaire was used to collect each participant’s social-demographic characteristics and lifestyle factors, including age, gender, marital status (married or others), educational status (primary school and lower, junior high school, senior high school, college and above), work intensity (light, moderate, vigorous, retirement), active smoking (never, occasional, frequent), passive smoking (yes or no), alcohol drinking (yes or no), fresh vegetable and fruit intake (< once/day or ≥ once/day). Leisure-time physical activity (LTPA) was assessed by a modified Global Physical Activity Questionnaire and the total volume of LTPA was calculated according to the method we reported [7, 22]. Personal history of chronic diseases, including hypertension, dyslipidemia, diabetes, chronic obstructive pulmonary disease (COPD), and cardiovascular diseases (CVDs) was collected. Hypertension was confirmed if the participant reported having physician-diagnosed hypertension, and/or had a mean systolic blood pressure (SBP) ≥ 140 mmHg, and/or mean diastolic blood pressure (DBP) ≥ 90 mmHg, and/or was on anti-hypertensive drugs [29]. A participant would be defined as dyslipidemia if he/she self-reported dyslipidemia diagnosed by a physician, or had serum cholesterol ≥ 5.2 mmol/l, LDC-CH ≥ 3.4 mmol/l, or TG ≥ 1.7 mmol/l [30]. A participant who had fasting plasma glucose ≥ 7.0 mmol/l and/or HbA1c ≥ 6.5%, and/or self-reported physician-diagnosed diabetes, and/or on diabetes treatment would be defined as having diabetes [31]. Height and weight were measured to calculate body mass index (BMI). Waist circumference and hip circumference were measured to calculate the waist-hip ratio (WHR). In addition, individual exposure to PM_2.5_ was assessed by 4-year average PM_2.5_ concentration from 2014 to 2017 within a 1,000 m circular buffer of each participant’s residential address. We obtained daily average PM_2.5_ data from 142 monitoring stations within or around Guangzhou City by the inverse distance weighting interpolation method [32].

### Statistical analysis

The Kolmogorov–Smirnov test was used to test for normality. Continuous variables that were normally distributed are presented as mean and standard deviation (SD), otherwise were presented as median and interquartile range (IQR). Distributions of categorical variables were presented as frequencies and percentages. The distribution of demographic and socioeconomic characteristics, history of chronic diseases, and glycolipid biomarkers were described. Comparison of characteristics among the non-OSA group, the pre-OSA group, and the OSA group, was conducted by one-way analysis of variance (ANOVA) for continuous variable and Chi-square tests for categorical variable. Each glycolipid biomarker was transformed into a categorical variable by using quartile methods. Unadjusted and adjusted odds ratios (ORs) with 95% confidence intervals (CIs) were estimated by using multivariate logistic regression models to display the association between glycolipid biomarkers and the risk of OSA and pre-OSA. The linear exposure-response relationship was examined by putting the median of each quartile of exposure as a continuous variable in the model. Sensitivity analysis was carried out by excluding participants with COPD and CVDs. The associations between dyslipidemia and diabetes and risk of the OSA and pre-OSA were also assessed among overall participants and participants without COPD and CVDs, as dyslipidemia and diabetes were diseases resulting from dysfunction of glycolipids. All analyses were performed using the R software (version 3.6.1). All tests were two-sided and a *p*-value of less than 0.05 was considered to be statistically significant.

## Results

Of the 10,826 participants included, 15.56% were categorized into the pre-OSA group, and 8.22% into the OSA group (Table 1). The proportion of males in the OSA group was 76.18% and was the highest among the three groups. Compared with participants without OSA, those with OSA were older and more likely to smoke frequently and drink alcohol, had higher levels of BMI [26.68 (3.98) kg/m^2^] and the waist-hip ratio [0.93 (0.06)], had a lower volume of LTPA [36.84 (34.77) MET-hours/week] and had a higher prevalence of dyslipidemia (77.19%) and diabetes (12.13%). The mean concentration of HDL-CH significantly decreased from 1.54 (0.43) mmol/L in the non-OSA group to 1.44 (0.41) mmol/L in the pre-OSA group and 1.39 (SD: 0.41) mmol/L in the OSA group. In contrast, the mean levels of LDL-CH, Triglyceride, and FBG increased in sequence from the non-OSA group, pre-OSA group, and OSA group (all *p* < 0.05).

**Table 1:** Basic characteristics of the participants.

Items	Non-OSA group	Pre-OSA group	OSA group	*p* value
Sample, *N* (%)	8,252 (76.22)	1,684 (15.56)	890 (8.22)	--
Age, year, mean (S.D.)	55.47 (10.14)	59.33 (8.73)	59.55 (8.36)	<0.001^*^
Body mass index, kg/m^2^, mean (S.D.)	23.44 (3.22)	25.43 (3.68)	26.68 (3.98)	<0.001^*^
Waist-hip ratio, mean (S.D.)	0.87 (0.07)	0.91 (0.06)	0.93 (0.06)	<0.001^*^
LTPA, MET-hours/week, mean (S.D.)	43.89 (35.90)	37.28 (32.07)	36.84 (34.77)	<0.001^*^
PM_2.5_ concentration, μg/m^3^, mean (S.D.)	49.21 (0.87)	49.18 (0.89)	49.16 (0.91)	0.0786^*^
Gender, *N* (%)				<0.001^†^
Male	1915 (23.21)	1,148 (68.17)	678 (76.18)	
Female	6,337 (76.79)	536 (31.83)	212 (23.82)	
Marital status, marital, *N* (%)				<0.001^†^
Married	7,156 (86.72)	1,547 (91.86)	825 (92.70)	
Others	1,096 (13.28)	137 (8.14)	65 (7.30)	
Educational status, mean (S.D.)				0.013^†^
Primary school and lower	2,970 (35.99)	600 (35.63)	308 (34.61)	
Junior high school	2094 (25.38)	437 (25.95)	204 (22.92)	
Senior high school	2086 (25.28)	416 (24.70)	276 (31.01)	
College and above	1,102 (13.35)	231 (13.72)	102 (11.46)	
Active smoking, *N* (%)				<0.001^†^
Never	7,055 (85.49)	1,002 (59.5)	485 (54.49)	
Occasional	257 (3.11)	189 (11.22)	131 (14.72)	
Frequent	940 (11.39)	493 (29.28)	274 (30.79)	
Passive smoking, *N* (%)				<0.001^†^
Yes	3,120 (37.81)	615 (36.52)	367 (41.24)	
No	5,132 (62.19)	1,069 (63.48)	523 (58.76)	
Alcohol drinking, *N* (%)				<0.001^†^
Yes	1,538 (18.64)	545 (32.36)	328 (36.85)	
No	6,714 (81.36)	1,139 (67.64)	562 (63.15)	
Work intensity, *N* (%)				<0.001^†^
Light	2,789 (33.80)	466 (27.67)	233 (26.18)	
Moderate	855 (10.36)	186 (11.05)	95 (10.67)	
Vigorous	448 (5.43)	84 (4.99)	55 (6.18)	
Retirement	4,160 (50.41)	948 (56.29)	507 (56.97)	
Fresh vegetable intake, *N* (%)				<0.001^†^
< once/day	107 (1.30)	43 (2.55)	25 (2.81)	
≥ once/day	8,145 (98.70)	1,641 (97.45)	865 (97.19)	
Fruit intake, *N* (%)				<0.001^†^
< once/day	1,594 (19.32)	390 (23.16)	267 (30.00)	
≥ once/day	6,658 (80.68)	1,294 (76.84)	623 (70.00)	
Hypertension, *N* (%)				<0.001^†^
Yes	1,375 (16.66)	972 (57.72)	821 (92.25)	
No	6,877 (83.34)	712 (42.28)	69 (7.75)	
Dyslipidemia, *N* (%)				<0.001^†^
Yes	5,673 (68.75)	1,219 (72.39)	687 (77.19)	
No	2,579 (31.25)	465 (27.61)	203 (22.81)	
Diabetes, *N* (%)				<0.001^†^
Yes	603 (7.31)	159 (9.44)	108 (12.13)	
No	7,649 (92.69)	1,525 (90.56)	782 (87.87)	
COPD, *N* (%)				0.008^†^
Yes	7,870 (95.37)	1,577 (93.65)	840 (94.38)	
No	382 (4.63)	107 (6.35)	50 (5.62)	
CVDs, *N* (%)				<0.001^†^
Yes	7,868 (95.35)	1,538 (91.33)	805 (90.45)	
No	384 (4.65)	146 (8.67)	85 (9.55)	
HDL-CH, mmol/L, mean (S.D.)	1.54 (0.43)	1.44 (0.41)	1.39 (0.41)	<0.001^*^
LDL-CH, mmol/L, mean (S.D.)	3.62 (0.99)	3.65 (0.97)	3.69 (0.96)	0.034^*^
Cholesterol, mmol/L, mean (S.D.)	5.47 (1.10)	5.47 (1.07)	5.54 (1.08)	0.065^*^
Triglyceride, mmol/L, mean (S.D.)	1.62 (1.40)	1.82 (1.48)	1.99 (1.79)	<0.001^*^
Fasting blood glucose, mmol/L, mean (S.D.)	5.49 (1.47)	5.71 (1.72)	5.88 (1.90)	<0.001^*^

* *p* value from one-way analysis of variance;

† *p* value from Chi-square test.

LTPA, leisure-time physical activity; PM2.5, atmospheric particulate matter (PM) with the diameter of less than 2.5; CVDs, cardiovascular diseases; COPD, chronic obstructive pulmonary disease; OSA, obstructive sleep apnea.

The associations between glycolipid biomarkers and the risk of pre-OSA and OSA were presented in Table 2. Every 1 mmol/l increment of HDL-CH was associated with a decreased risk of both pre-OSA (OR: 0.82, 95% CI: 0.70–0.96) and OSA (OR: 0.63, 95% CI: 0.50–0.79) after adjustment for covariates. Compared with subjects within the lowest quartile of HDL-CH, the adjusted OR of pre-OSA and OSA in subjects within the highest quartile (>1.76 mmol/l) was 0.78 (95% CI: 0.65–0.94) and 0.59 (95% CI, 0.45–0.78), respectively; and a significant exposure-response trend was observed for both pre-OSA and OSA (both *p* < 0.05).

**Table 2:** Association between glycolipid biomarkers and the risk of obstructive sleep apnea.

	Sample size			Crude OR (95% CI)		Adjusted OR (95% CI)^*^	
	Non-OSA	Pre-OSA	OSA	Pre-OSA vs. Non-OSA	OSA vs. Non-OSA	Pre-OSA vs. Non-OSA	OSA vs. Non-OSA
HDL-CH							
Quartile 1 (≤ 1.21)	1943	517	332	1.00	1.00	1.00	1.00
Quartile 2 (> 1.21 ~ ≤ 1.46)	2004	450	231	0.84 (0.73, 0.97)	0.68 (0.56, 0.81)	0.96 (0.81, 1.14)	0.79 (0.62, 1.01)
Quartile 3 (> 1.46 ~ ≤ 1.76)	2,118	406	181	0.72 (0.62, 0.83)	0.50 (0.41, 0.61)	0.97 (0.81, 1.15)	0.70 (0.54, 0.91)
Quartile 4 (> 1.76)	2,187	311	146	0.53 (0.46, 0.62)	0.39 (0.32, 0.48)	0.78 (0.65, 0.94)	0.59 (0.45, 0.78)
*P* for trend				< 0.001	< 0.001	0.019	< 0.001
Every 1 mmol/l increment				0.58 (0.51, 0.66)	0.42 (0.35, 0.50)	0.82 (0.70, 0.96)	0.63 (0.50, 0.79)
LDL-CH							
Quartile 1 (≤ 2.95)	2,106	408	201	1.00	1.00	1.00	1.00
Quartile 2 (> 2.95 ~ ≤3.56)	2,104	409	219	1 (0.86, 1.17)	1.09 (0.89, 1.33)	1.11 (0.92, 1.34)	1.19 (0.91, 1.57)
Quartile 3 (> 3.56 ~ ≤4.24)	2037	422	239	1.07 (0.92, 1.24)	1.23 (1.01, 1.5)	1.14 (0.92, 1.43)	1.18 (0.86, 1.62)
Quartile 4 (> 4.24)	2005	445	231	1.15 (0.99, 1.33)	1.21 (0.99, 1.47)	1.13 (0.90, 1.41)	0.98 (0.72, 1.35)
*P* for trend				0.0456	0.0335	0.360	0.684
Every 1 mmol/l increment				1.03 (0.98, 1.09)	1.07 (0.99, 1.15)	1.01 (0.93, 1.09)	0.99 (0.89, 1.10)
Total cholesterol							
Quartile 1 (> 4.72)	2083	428	196	1.00	1.00	1.00	1.00
Quartile 2 (> 4.72 ~ ≤ 5.39)	2083	407	234	0.95 (0.82, 1.10)	1.19 (0.98, 1.46)	0.94 (0.77, 1.14)	1.09 (0.82, 1.45)
Quartile 3 (> 5.39 ~ ≤ 6.12)	2068	414	220	0.97 (0.84, 1.13)	1.13 (0.92, 1.38)	0.96 (0.76, 1.21)	0.93 (0.66, 1.30)
Quartile 4 (> 6.12)	2018	435	240	1.05 (0.91, 1.22)	1.26 (1.04, 1.54)	0.93 (0.74, 1.18)	0.89 (0.64, 1.24)
*P* for trend				0.478	0.047	0.625	0.295
Every 1 mmol/l increment				1 (0.96, 1.05)	1.06 (0.99, 1.13)	0.98 (0.91, 1.05)	0.97 (0.88, 1.07)
Triglyceride							
Quartile 1 (≤ 0.95)	2,218	348	150	1.00	1.00	1.00	1.00
Quartile 2 (> 0.95 ~ ≤ 1.34)	2,140	391	187	1.17 (1.01, 1.36)	1.29 (1.03, 1.62)	1.04 (0.86, 1.25)	1.02 (0.76, 1.37)
Quartile 3 (> 1.34 ~ ≤ 1.95)	2003	433	263	1.38 (1.18, 1.61)	1.94 (1.57, 2.39)	1.22 (1.01, 1.47)	1.41 (1.05, 1.89)
Quartile 4 (> 1.95)	1891	512	290	1.73 (1.49, 2.00)	2.27 (1.84, 2.79)	1.32 (1.08, 1.60)	1.56 (1.18, 2.07)
*P* for trend				< 0.001	< 0.001	0.002	0.002
Every 1 mmol/l increment				1.10 (1.07, 1.14)	1.15 (1.10, 1.19)	1.01 (0.98, 1.05)	1.03 (0.98, 1.09)
Fasting blood glucose							
Q1 (≤ 4.84)	2,256	345	166	1.00	1.00	1.00	1.00
Q2 (> 4.84 ~ ≤ 5.18)	2085	391	179	1.23 (1.05, 1.43)	1.17 (0.94, 1.45)	1.11 (0.92, 1.34)	1.01 (0.76, 1.35)
Q3 (> 5.18 ~ ≤ 5.68)	2031	441	250	1.42 (1.22, 1.66)	1.67 (1.36, 2.05)	1.07 (0.89, 1.28)	1.13 (0.86, 1.48)
Q4 (> 5.68)	1880	507	295	1.76 (1.52, 2.05)	2.13 (1.75, 2.60)	1.37 (1.13, 1.67)	1.38 (1.03, 1.85)
*P* for trend				< 0.001	< 0.001	0.006	0.022
Every 1 mmol/l increment				1.09 (1.06, 1.12)	1.14 (1.10, 1.18)	1.05 (0.99, 1.11)	1.03 (0.96, 1.12)

* Adjustment for age, gender, marital status, educational status, body mass index, leisure-time physical activity, waist-hip ratio, PM_2.5_ concentration, work intensity, active smoking, passive smoke, alcohol drinking, fruit intake, fresh vegetable intake, hypertension, dyslipidemia, diabetes, chronic obstructive pulmonary disease, and cardiovascular diseases.

HDL-CH, high-density lipoprotein cholesterol; LDC-CH, low-density lipoprotein cholesterol; OSA, obstructive sleep apnea; FBG, obstructive sleep apnea.

Conversely, when comparing the highest with the lowest quartile, triglyceride was associated with a 32% (OR: 1.32, 95% CI: 1.08–1.60) and a 41% (OR: 1.59, 95% CI: 1.18–2.07) increased risk of pre-OSA and OSA, separately; FBG was associated with a 37% (OR: 1.37, 95% CI: 1.13–1.67) and a 38% (OR: 1.38, 95% CI: 1.03–1.85) increased risk of pre-OSA and OSA, separately; significant exposure-response trends were also observed for pre-OSA and OSA (all *p* < 0.05). However, every 1 mmol/l increment of Triglyceride or FBG was associated with a slightly increased risk of pre-OSA and OSA, despite that the associations were nonsignificant. No significant association of hemoglobin, LDL-CH, or cholesterol with the risk of both pre-OSA and OSA was observed.

Besides, subjects with dyslipidemia had a higher risk of pre-OSA (OR 1.19, 95% CI 1.06–1.34) and OSA (OR 1.54, 95% CI 1.31–1.81) in the crude model. However, the association disappeared after adjusting for covariates (Table 3). Similarly, there was no significant association between diabetes and the risk of pre-OSA (OR 1.08, 95% CI 0.87–1.25) or OSA (OR 1.29, 95% CI 0.96–1.74) after adjustment for covariates.

**Table 3:** Association between dyslipidemia and diabetes and the risk of obstructive sleep apnea.

	Crude OR (95% CI)		Adjusted OR (95% CI)^*^	
	Pre-OSA vs. Non-OSA	OSA vs. Non-OSA	Pre-OSA vs. Non-OSA	OSA vs. Non-OSA
Dyslipidemia				
No	1.00	1.00	1.00	1.00
Yes	1.19 (1.06, 1.34)	1.54 (1.31, 1.81)	1.00 (0.86, 1.14)	1.11 (0.90, 1.38)
Diabetes				
No	1.00	1.00	1.00	1.00
Yes	1.32 (1.10, 1.59)	1.75 (1.41, 2.18)	1.08 (0.87, 1.25)	1.29 (0.96, 1.74)

* Adjustment for age, gender, marital status, educational status, body mass index, leisure-time physical activity, waist-hip ratio, PM_2.5_ concentration, work intensity, active smoking, passive smoke, alcohol drinking, fruit intake, fresh vegetable intake, hypertension, dyslipidemia, diabetes, chronic obstructive pulmonary disease, and cardiovascular diseases.

OSA, obstructive sleep apnea.

The sensitivity analysis was conducted by excluding participants with COPD and CVDs, and similar results were obtained between five glycolipid biomarkers and risk of Pre-OSA and OSA (Table 4), and between dyslipidemia and diabetes and risk of Pre-OSA and OSA (Table 5).

**Table 4:** Association between glycolipid biomarkers and OSA risk after excluding participants with COPD and CVDs.

	Sample size			Adjusted OR (95% CI)^*^	
	Non-OSA	Pre-OSA	OSA	Pre-OSA vs. Non-OSA	OSA vs. Non-OSA
HDL-CH					
Quartile 1 (≤ 1.21)	1748	461	284	1.00	1.00
Quartile 2 (> 1.21 ~ ≤ 1.46)	1812	372	206	0.89 (0.74, 1.07)	0.81 (0.63, 1.04)
Quartile 3 (> 1.46 ~ ≤ 1.76)	1946	345	153	0.92 (0.76, 1.11)	0.70 (0.53, 0.92)
Quartile 4 (> 1.76)	2010	273	123	0.76 (0.62, 0.93)	0.57 (0.42, 0.76)
*P* for trend				0.014	<0.001
Every 1 mmol/l increment				0.82 (0.69, 0.96)	0.61 (0.47, 0.78)
LDL-CH					
Quartile 1 (≤ 2.95)	1904	343	170	1.00	1.00
Quartile 2 (> 2.95 ~ ≤ 3.56)	1905	357	190	1.12 (0.92, 1.36)	1.14 (0.85, 1.53)
Quartile 3 (> 3.56 ~ ≤ 4.24)	1873	370	205	1.12 (0.88, 1.43)	1.07 (0.76, 1.50)
Quartile 4 (> 4.24)	1834	381	201	1.10 (0.87, 1.40)	0.93 (0.66, 1.30)
*P* for trend			*NA*	0.529	0.501
Every1 mmol/l increment				1.00 (0.92, 1.09)	0.98 (0.88, 1.1)
Cholesterol					
Quartile 1 (> 4.72)	1877	365	163	1.00	1.00
Quartile 2 (> 4.72 ~ ≤ 5.39)	1888	348	208	0.89 (0.72, 1.09)	1.01 (0.75, 1.37)
Quartile 3 (> 5.39 ~ ≤ 6.12)	1909	367	183	0.93 (0.72, 1.19)	0.79 (0.56, 1.14)
Quartile 4 (> 6.12)	1842	371	212	0.87 (0.68, 1.12)	0.83 (0.58, 1.18)
*P* for trend				0.387	0.184
Every 1 mmol/l increment				0.96 (0.89, 1.03)	0.95 (0.86, 1.06)
Triglyceride					
Quartile 1 (≤ 0.95)	2029	293	130	1.00	1.00
Quartile 2 (> 0.95 ~ ≤ 1.34)	1933	337	157	1.09 (0.89, 1.34)	1.02 (0.75, 1.40)
Quartile 3 (> 1.34 ~ ≤ 1.95)	1829	378	222	1.29 (1.05, 1.58)	1.58 (1.17, 2.14)
Quartile 4 (> 1.95)	1725	443	257	1.32 (1.07, 1.63)	1.42 (1.04, 1.94)
*P* for trend				0.003	0.003
Every 1 mmol/l increment				1.00 (0.96, 1.05)	1.03 (0.98, 1.09)
Fasting blood glucose (FBG)					
Q1 (≤ 4.84)	2061	296	146	1.00	1.00
Q2 (> 4.84 ~ ≤ 5.18)	1904	324	151	1.08 (0.89, 1.32)	0.98 (0.72, 1.33)
Q3 (> 5.18 ~ ≤ 5.68)	1849	396	215	1.15 (0.95, 1.40)	1.14 (0.86, 1.52)
Q4 (> 5.68)	1702	435	254	1.34 (1.08, 1.66)	1.31 (0.96, 1.78)
*P* for trend				0.006	0.054
Every 1 mmol/L increment				1.04 (0.98, 1.11)	1.03 (0.95, 1.11)

* Adjustment for age, gender, marital status, educational status, body mass index, leisure-time physical activity, waist-hip ratio, PM_2.5_ concentration, work intensity, active smoking, passive smoke, alcohol drinking, fruit intake, fresh vegetable intake, hypertension, dyslipidemia, diabetes, chronic obstructive pulmonary disease, and cardiovascular diseases.

HDL-CH, high-density lipoprotein cholesterol; LDC-CH, low-density lipoprotein cholesterol; OSA, obstructive sleep apnea; FBG, obstructive sleep apnea; CVDs, cardiovascular diseases; COPD, chronic obstructive pulmonary disease.

**Table 5:** Association between dyslipidemia and diabetes and risk of pre-OSA and OSA after excluding participants with COPD and CVDs.

	Sample size			Crude OR (95% CI)		Adjusted OR (95% CI)^*^	
	Non-OSA	Pre-OSA	OSA	Pre-OSA vs. Non-OSA	OSA vs. Non-OSA	Pre-OSA vs. Non-OSA	OSA vs. Non-OSA
Dyslipidemia							
No	2,344	398	169	1.00	1.00	1.00	1.00
Yes	5,172	1,053	597	1.2 (1.06, 1.36)	1.60 (1.34, 1.91)	0.96 (0.82, 1.11)	1.08 (0.86, 1.36)
Diabetes							
No	6,974	1,310	671	1.00	1.00	1.00	1.00
Yes	574	141	95	1.38 (1.14, 1.68)	1.82 (1.45, 2.30)	1.12 (0.88, 1.41)	1.27 (0.93, 1.74)

* Adjustment for age, gender, marital status, educational status, body mass index, leisure-time physical activity, waist-hip ratio, PM_2.5_ concentration, work intensity, active smoking, passive smoke, alcohol drinking, fruit intake, fresh vegetable intake, hypertension, diabetes (only for association with dyslipidemia) and for dyslipidemia (only for association with diabetes).

OSA, obstructive sleep apnea; CVDs, cardiovascular diseases; COPD, chronic obstructive pulmonary disease.

## Discussion

To our best knowledge, this is the first study to assess the association between glycolipid biomarkers and the risk of OSA identified by the combination of the Berlin Questionnaire and STOP-BANG Questionnaire in China. Given the difficulty of OSA diagnosis by polysomnography in a large-scale population [33], the use of double questionnaires could improve the specificity of OSA. This present study found that a higher level of HDL-CH was associated with a decreased risk of both pre-OSA and OSA, while triglyceride and FBG were positively associated with the risk of both pre-OSA and OSA. No significant association of LDL-CH, cholesterol, dyslipidemia, or diabetes with the risk of both pre-OSA and OSA was observed.

Metabolic abnormalities could increase the chance of upper airway collapsibility [8, 34]. Previous studies used serum lipid parameters to predict the risk of OSA, such as glucose [35], TG [9], and HDL-CH [14]. Novel composite parameters of glycolipids such as lipid accumulation product (LAP) also showed strong associations with OSA [36]. Some clinical OSA cohorts reported a positive association between TG and AHI [9, 37]. Moreover, a twin study analyzed the heritability of the relationship between OSA and hypertriglyceridemia and found that common genetic factors significantly determined the relationship between indices of chronic intermittent hypoxia and serum TG levels [38]. A genome-wide association study on the United Kingdom Biobank also indicated that genetically increased TG levels have independent causal effects on the risk of sleep apnea without the confounding effects of obesity [39]. Consistently, our study found similar results that triglyceride and fasting blood glucose were positively associated with the risk of both OSA and pre-OSA, whereas HDL-CH was negatively associated with the risk of both OSA and pre-OSA, further suggesting that regardless of the number of OSA-related symptoms or severity of OSA, glycolipid biomarkers may help determine risk and should be controlled during daily life. In this study, we took both BMI and WHR into consideration to exclude the confounding effect of peripheral and abdominal adiposity, which indicated that elevated level of HDL and TG was independently associated with OSA risk. Compared with peripheral obesity, abdominal obesity has a greater effect on upper airway function [40, 41].

In adult individuals, a large number of cross-sectional studies have shown independent associations between fasting levels of TC and the severity of OSA, particularly the frequency of intermittent hypoxic events [34]. Evidence from a Chinese large-scale cross-sectional study showed that, of the various components in serum lipid, only LDL-CH was independently associated with OSA [42]. However, we did not find a significant association of TC or LDL-CH with OSA risk. Furthermore, the results from our study were opposite to results from the ELSA-Brazil cohort study that drew the conclusion that OSA was independently associated with total cholesterol but not with HDL levels [37]. The conflicts may be connected with the reason that the possible association of TC and LDL-CH with OSA can be covered by comorbidities of OSA, such as hypertension and multiple cardiovascular diseases, due to their shared risk factors [34, 43]. Moreover, evidence showed that oxidized LDL (oxLDL) was associated with OSA. OxLDL is no longer recognized by cellular receptors with consequent inflammation and plaque formation on the internal surfaces of blood vessels [44]. This could be due to the fact that LDL particles are not removed by the liver and peripheral cells due to the depletion of LDL receptor-related protein-1 (LRP-1) in OSA [45]. In addition, emerging randomized trials for patients with OSA found that plasma levels of lipid biomarkers were reversed by CPAP treatment, which suggests causality [34]. Taking these elements together, we found that pre-OSA and OSA were associated with HDL and TG levels, but not with dyslipidemia. The possible explanation may be that even a minor change of blood lipid can initiate the pathologic development of OSA, and this change does not necessarily depend on the presence of specific symptoms, whereas dyslipidemia is widely believed to be closely associated with OSA [8, 9].

Noticeably, we did not find any association of diabetes with pre-OSA risk and OSA risk. Nevertheless, we found that the highest quantile of blood glucose (> 5.68 mmol/l) was linked with an increased risk of pre-OSA and OSA. Although diabetes was commonly assessed by fasting blood glucose higher than ≥7.0 mmol/l, our findings suggested that even a lower level of FBG could lead to a higher risk of pre-OSA and OSA. OSA is commonly considered as frequent comorbidity in patients with type 2 diabetes, and cardinal features of OSA, including intermittent hypoxemia and sleep fragmentation, have been linked to abnormal glucose metabolism in laboratory-based experiments [46]. The relationship between OSA and type 2 diabetes may be bidirectional in nature given that diabetic neuropathy can affect the central control of respiration and upper airway neural reflexes, promoting sleep-disordered breathing [46]. Early attention to individual blood glucose levels may have significant preventive implications for reducing OSA prevalence.

Our study has some strengths. First, we conjunctively used the Berlin Questionnaire and the STOP-BANG Questionnaire, two widely used questionnaires with high validity and reliability, to identify OSA statuses, which enhanced the screening specificity. Second, we studied the association between several glycolipid biomarkers and the risk of pre-OSA and OSA among a representative population in Guangdong, which attenuated selection bias. Third, we took a large number of confounding factors into account, including PM_2.5_ exposure, individual lifestyles, and abdominal adiposity indicator, which could eliminate confounders to a large degree and reveal the independent associations between those indicators and OSA. Finally, sensitivity analyses yielded similar results, demonstrating the robustness of our findings.

However, there are also some limitations. First, we used questionnaires to define OSA rather than overnight polysomnography, for it was difficult to implement in a large-scale population study. Nevertheless, the questionnaires used in this study showed high validity and reliability in predicting OSA [26, 27] and were commonly implemented in previous studies [26, 27, 47]. Second, the nature of a cross-sectional study could not support causality inference. Since OSA often coexists with various chronic diseases such as hyperlipidemia, diabetes, and cardiovascular disease, or acts as an intermediate link in the occurrence and development of these diseases, it is difficult to clarify the mechanism of glucose and lipid metabolism and OSA. Further studies with longitudinal design are needed to confirm this relationship.

## Conclusion

The findings suggest that the level of HDL-CH was inversely associated with OSA risk, while elevated serum triglyceride and FBG could increase the risk of OSA. Healthy glycolipid metabolism warrants more attention in the field of OSA prevention, and more reports from rigorous longitudinal studies are expected.

## Data availability statement

The raw data supporting the conclusions of this article will be made available by the authors, without undue reservation.

## Ethics statement

The studies involving human participants were reviewed and approved by the Ethical Review Committee for Biomedical Research, School of Public Health, Sun Yat-sen University, and Ethics Committee of Guangzhou Centre for Disease Control and Prevention. The patients/participants provided their written informed consent to participate in this study.

## Author contributions

XL and HD conceived and designed the study. HD, HZ, MZ, GS, JC, XW, SR, JH, and XL collected the data. MZ and HZ analyzed the data. MZ, XD, and WS drafted the manuscript. XL, HZ, HD, JH, and WZ reviewed and edited the manuscript. All authors contributed to the article and approved the submitted version.

## Funding

This work was supported by the Guangdong Basic and Applied Basic Research Foundation (No. 2022A1515010686), the Medical Science and Technology Research Foundation of Guangdong Province (No. A2023408), the Guangdong Provincial Key R&D Program (No.2019B020230004) and the National Key R&D Program of China (No.2018YFC1312502). The founder had no role in the design, analysis, or writing of this manuscript.

## Conflict of interest

The authors declare that the research was conducted in the absence of any commercial or financial relationships that could be construed as a potential conflict of interest.

## Publisher’s note

All claims expressed in this article are solely those of the authors and do not necessarily represent those of their affiliated organizations, or those of the publisher, the editors and the reviewers. Any product that may be evaluated in this article, or claim that may be made by its manufacturer, is not guaranteed or endorsed by the publisher.
